# Challenges in Wireless System Integration as Enablers for Indoor Context Aware Environments

**DOI:** 10.3390/s17071616

**Published:** 2017-07-12

**Authors:** Peio López-Iturri, Erik Aguirre, Leyre Azpilicueta, José Javier Astrain, Jesús Villandangos, Francisco Falcone

**Affiliations:** 1 Electrical and Electronic Engineering Department, Public University of Navarre, Pamplona 31006, Spain; peio.lopez@unavarra.es (P.L.-I.); erik.aguirre@unavarra.es (E.A.); 2 Institute of Smart Cities, Public University of Navarre, Pamplona 31006, Spain; josej.astrain@unavarra.es (J.J.A.); jesusv@unavarra.es (J.V.); 3 School of Engineering and Sciences, Tecnologico de Monterrey, Monterrey 64849, Mexico; leyre.azpilicueta@itesm.mx; 4 Mathematical Engineering and Computer Science Department, Public University of Navarre, Pamplona 31006, Spain

**Keywords:** smart cities, smart regions, context aware environments, wireless sensor networks (WSN), interference management

## Abstract

The advent of fully interactive environments within Smart Cities and Smart Regions requires the use of multiple wireless systems. In the case of user-device interaction, which finds multiple applications such as Ambient Assisted Living, Intelligent Transportation Systems or Smart Grids, among others, large amount of transceivers are employed in order to achieve anytime, anyplace and any device connectivity. The resulting combination of heterogeneous wireless network exhibits fundamental limitations derived from Coverage/Capacity relations, as a function of required Quality of Service parameters, required bit rate, energy restrictions and adaptive modulation and coding schemes. In this context, inherent transceiver density poses challenges in overall system operation, given by multiple node operation which increases overall interference levels. In this work, a deterministic based analysis applied to variable density wireless sensor network operation within complex indoor scenarios is presented, as a function of topological node distribution. The extensive analysis derives interference characterizations, both for conventional transceivers as well as wearables, which provide relevant information in terms of individual node configuration as well as complete network layout.

## 1. Introduction

The evolution towards Smart Cities and by extension, Smart Regions, is driving the need of increasing user-environment interactivity levels. In this sense, Context Aware environments are foreseen as key elements in the control and regulation of the multiple systems which sustain Smart Cities, such as Intelligent Transportation Systems, Smart Health, Smart Grids, context-based marketing or e/open-Government, to name but a few [1,2,3,4]. The benefits inherent to the use of Context Aware environments are mainly given by the reactive and in some instances proactive nature of these scenarios, in which multiple systems are capable of adapting to user needs as well as to the requirements of such systems. Large scale sensing capabilities as well as scalable communication networks with high mobility levels are compulsory in order to provide the required levels of interactivity within these environments. To this extent, multiple, massive transceiver deployments operating with a diverse array of communication system in a Heterogeneous Network mode are being considered in order to exploit the involved systems, as well as to provide a mechanism to gather and fuse data, which can be handled by upper-level business intelligence layers. Wireless communication systems spanning from Radio Frequency (RF) to millimeter wave spectrum are primarily adopted given their performance in terms of cost, size and coverage range. RF and microwave bands have been widely adopted, given the existence of multiple Industrial, Scientific and Medical bands, as well as by the massive deployment of Public Land Mobile Systems (mainly 3G and 4G) and wireless broadcast systems, such as radio and television services. In parallel, the progressive requirements in Internet-based connectivity given by the advent of the Internet of Things (IoT) renders forecasts in which by the next decade, the number of IP connected devices will increase by one to two orders of magnitude [5,6,7,8,9]. In order to comply with cost, energy and size restrictions, transceiver configurations and communication protocols must be kept simple and low cost. In parallel, requirements in relation with communication capabilities, in terms of bit rate, latency and Quality of Service (QoS) demands, must be met. Digital transmission systems employ adaptive modulation and coding schemes, coupled to other functionalities, such as time/space diversity or channel multiplexing, leading to time dependent communication channels. This later case requires the analysis of coverage/capacity relations, in order to guarantee adequate service values for overall communications (and hence, for the end systems within the Context Aware scenarios).

The variability in channel characterization is given by multiple factors, such as user service demands (which can be time-dependent, as a function of the multiple services under consideration), user density, user location and user location variability, among others. The limiting factor is given by the total amount of interference experienced by each one of the transceivers within the system, as coverage/capacity relations are directly degraded as a function of overall spectral density values of interference within the bandwidth of operation. 

The advent of IoT conveys a relevant growth in wireless transceiver density. This condition is particularly relevant in the case of indoor scenarios, in which a large number of devices are embedded within the scenario (e.g., building automation systems, location and tracking systems, communication backup for home entertainment systems, etc.) and other non-structural transceivers, such as wearables from the users within the indoor environment are also present. Moreover, indoor scenarios represent a relevant part of Context Aware environments, such as indoor vehicular communications within Intelligent Transportation Systems Page: 2 (ITS) (e.g., passenger trains, freight handling, in flight aircraft communications), Smart Health (eHealth, mHealth, Ambient Assisted Living), Context-based marketing and shopping, home and building automation systems, indoor tracking and location systems and many other applications inherent to the progressive adoption of Smart Cities/Smart Region paradigms. It is also worth noting that node density and distribution are of interest in assessing network performance and providing alternative network metrics and functionalities. In [10], the node concentration concept is presented, which considers the average number of neighbors connected to each node, given a specific transmission power. This is used in order to implement an enhance Ad-hoc On-demand Distance Vector protocol which employs a node concentration driven gossiping approach for limiting the flooding of the route request packets, which impact network performance. In [11], agent-modelled IoT networks of different scales are simulated, with the goal of analyzing issues and bottlenecks at communication level, showing the influence of node concentration and distribution. Impact of interference on wireless sensor networks has been analyzed in [12] showing that IEEE 802.11 and microwave ovens cause significant increases in Packet Error Rates, raising their values from a typical value of approximately 2% with no interference sources present to an upward of 25% depending upon the distances among the different nodes and interferer sources. Other aspects can be also considered, such as node distribution and the role of data acquisition in IoT enhanced environments, such as virtual agricultural systems [13]. Further considerations in WSN operation can be found in relation with operation in environments in which multiple data flows not only own different priorities but also have different levels of importance or criticality, leading to mixed criticality scenarios, which have been studied in the case of Wireless HART (Highway Addressable Remote Transducer Protocol) systems [14].

Analyzing the impact of interference is a complex issue, due to the variability and nature of the different interference sources. In general terms, interference can be considered non-correlated to the useful signals which are transmitted within the target transmitter. In this way, all interference sources (intra-system, inter-system and background noise) can be described in terms of additive noise, without loss of generality, enabling the selective introduction of potential interference sources. In this way, a dense array of transmitters has been considered, which can be activated as required in order to analyze multiple interference scenarios.

In this work, an extensive analysis of wireless sensor network performance within indoor scenarios emulating Context Aware applications will be described. In order to analyze potential performance metrics, precise scenario modelling and analysis will be derived by employing ad-hoc deterministic tools, in which complete scenario as well as network complexity will be considered. Different network densities and node distributions will be considered, as well as static (i.e., infrastructure) and variable (i.e., wearable) nodes within the scenario, in order to evaluate variations of interference spectral power densities and hence, degradation within performance metrics. 

## 2. Simulation Technique and Scenario under Analysis

Overall user experience within Context Aware environments is given by user interaction, with other users as well as with multiple autonomous systems, such as transportation, building automation or location and tracking, to name a few. In terms of communication systems, low latency levels are required (particularly in the case of real time services) and adequate transmission bit rates (which must adapt to variable user requirements). The advent of Context Aware environments is mainly foreseen by the adoption of IoT (enabling connectivity among any potential device and application) and the consolidation of 5G networks, in particular in the case of Device to Device (D2D) connectivity [5,6,7,8,9]. These scenarios are of particular interest in the case of high user/device density, particularly in confined environments, given the large number of potential users (e.g., office spaces, shopping centers, etc.) and devices which require communication capabilities. These results are determined not only by user location and density, but also by dynamic requirements in QoS levels, such as transmission rate, bit error rate or latency, among others. 

The analysis of confined scenarios is a complex task, given inherent complexity in terms of object density and material parameters, as well as in user location variability, particularly in the case of wearable devices. In terms of intra-device and device to concentrator links (which is the case to consider when devices require intermediate hubs, such as wireless Local Area Network (LAN) access points or coordinators in 802.15 networks), radio link analysis is compulsory in order to estimate device operation as a function of available transmitter power, receiver sensitivity and overall interference levels. Due to the large number of devices and the interaction of radio waves with the surrounding environment, precise characterization is a computationally demanding task. Multiple simulation techniques can be employed, such as empirical based ones, in which analytical expressions are obtained based on regression methods and curve fitting. Multiple models have been proposed, as a function of surrounding environment and the operational range employed (e.g., frequency of operation, transceiver heights, building heights, etc.), such as Okumura-Hata, Xia-Bertoni, COST-231 or one slope, dual slope models, to name a few [15,16,17,18,19,20]. These models exhibit low computational complexity but are strongly site specific, requiring intensive measurement campaigns in order to adapt the model to the specific scenarios under analysis. Moreover, effects such as diffuse scattering or multi-obstacle diffraction must be considered separately. 

On the other hand, deterministic-based methods directly solve Maxwell equations in every region of space. Several approaches can be followed in this case: from full wave electromagnetic simulation techniques, such as Finite Difference Time Domain, Finite Integration Time Domain or Method of Moments, to model simplifications based on geometric optics. These methods are more precise and are, in principle, non-site specific. However, their computational cost is much higher than in the case of analytical based approaches. An adequate balance between computational cost and accuracy, for the case of large scenarios (as compared with operational wavelength) in which wireless channel must be analyzed can be obtained by employing ray tracing and ray launching techniques. In this work, an ad-hoc implemented simulation tool based on 3D Ray Launching will be employed in order to analyze the behavior of wireless sensor network operation as a function of node density, transceiver specifications and scenario characteristics. The simulation method has been extensively tested in a number of different applications related with context aware environments, such as Intelligent Transportation systems, Smart Health, and sports, to name a few [21,22,23,24,25].

**Figure 1:**
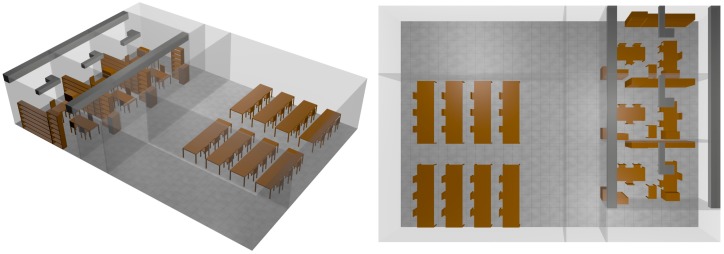
Schematic representation of the simulation scenario, corresponding to an indoor office space, with different work zones.

In order to perform the proposed network node density variation analysis, a complex indoor scenario has been employed, which is schematically depicted in Figure 1. It corresponds with a real location within the university campus at Universidad Pública de Navarra (UPNA). The scenario includes different zones, with office spaces, common transit zones and a functional lab. Depending on the zone under consideration, node density will vary, as a function of infrastructure node locations as well as on the presence of wearable devices carried by users. The complete furnishing of the office space has been considered, in terms of dimensions and locations. Dispersive material parameters as a function of the corresponding frequency of operation have also been considered, which are depicted in Table 1 and Table 2.

The 3D Ray Launching (3D RL) simulation technique is based on the introduction of transmitter sources which emulate radio propagation by discretizing the wave front in a series of launched rays, which follow the same direction as the propagating wave vector in free space. The methodology is based on the approximation of electromagnetic field propagation and interaction with the surrounding media in terms of geometric optics combined with Uniform Theory of Diffraction, which has been extensively documented [25,26,27,28,29,30], considering phenomena such as as wave reflection, transmission and refraction, as well as equivalent losses owing to diffracted fields in edges.

The simulation method employed has been implemented ad-hoc, taking into consideration a volumetric spatial mapping of the scenario under analysis, by the construction of a discretized mesh of cuboids, which can be mapped by zones into different sizes, depending on the specific simulation requirements. Relevant parameters in simulation are related with angular resolution, cuboid size and maximum number of reflected components in relation with the main propagating specular component. These parameters have been chosen in accordance with convergence analysis studies applied to the specific scenario under analysis in this work [27]. The 3D RL code has been optimized in order to enable hybrid simulation approaches, based on 3D RL + Neural Network interpolators, 3D RL + Electromagnetic Diffusion equation as a function of the bidimensional density of duffractive elements or 3D RL + Collaborative Filtering techniques, in order to reduce computational complexity and hence enable simulation of large complex scenarios. Moreover, ad-hoc human body models with scalable parameters (material complexity and body dimensions) have been implemented in order to enable their inclusion in the 3D RL code. In this way, user interaction as well as consideration of wearable devices is feasable. The set of parameters employed in the simulation scenarios along this work are shown in Table 3. 

## 3. Wireless Sensor Network Radioelectric Analysis

As previously stated, one of the main considerations in order to provide assessment on context aware system performance is to obtain coverage/capacity estimations. The proposed increase in transceiver density inherent to connectivity requirements to provide interactive environments gives also rise to potential increase in interference values, as well as a complex received power level profiles, which are dependent on the configuration of the elements within the scenario under analysis. In this sense and taking into consideration requirements of IoT, networks based on multiple and heterogeneous transceivers, such as Narrowband LTE (NB-LTE), 5G Device to Device (5G D2D), wireless personal area networks and wireless local area networks are employed. This leads to a large, non-uniform set of transceivers, with multiple transmission schemes, in which interference as well as energy control play key roles in overall system performance [31,32]. Absoulte radio channel levels are strongly dependent in the case of indoor scenarios (in which non line of sight communications coexist with line of sight links) of elements such as furnishings, materials or user locations, among others [32]. 

Different approaches can be followed to perform wireless channel characterization, many of them based on simplified empirical based propagation models, such as one slope, dual slope, COST 231 multi wall, etc. [32]. In this case, path loss estimations can be obtained in a straightforward manner in large scenarios. The main drawback following these approaches is oversimplification, in terms of homogeneous transceiver consideration (i.e., all of the transceivers within the scenario are parameterized in the same way, owing to inherent restrictions in terms of the employed models) as well as on the interaction with the surrounding environment, in terms of fast fading components given by multipath propagation. 

On the other hand, full wave simulation techniques such as Finite Difference Time Domain or Method of Moments can be employed in order to explicitly solve Maxwell equations in the simulation scenario. These methods provide precise results, but require large computational resources, specially in the case of increasing the frequency of operation. In the case of IoT indoor scenarios, the use of wireless transceivers in the microwave spectrum (mainly 2.4 GHz and 5.8 GHz) is compulsory, in order to consider service provision between devices (D2D communication schemes in the case of 5G networks or Machine to Machine, M2M in the case of 3G or 4G networks), between users (considering mobile terminals as well as wearables) or between service gateways and infrastructure nodes (for example in the case of access points in Wireless Local Area Networks or aggregators of Wireless Sensor Networks). Taking into consideration the large number of potential transceivers located within indoor context aware environments, with values spanning from 1 transceiver/100 m^2^ to (1–2) transceiver/1 m^2^ (which can increase in the case of specific body area networks) and the fact that wireless channel characterization is the prime element in system level analysis (i.e., to obtain Key Performance Indicators in order to evaluate QoS), alternative methods must be sought. As stated in Section 2, the approach followed is to employ the 3D Ray Launching technique, which is based on the approximation of the impinging wavefront given by a discrete set of rays, following an approximation given by geometric optics and Uniform Theory of Diffraction.

**Figure 2:**
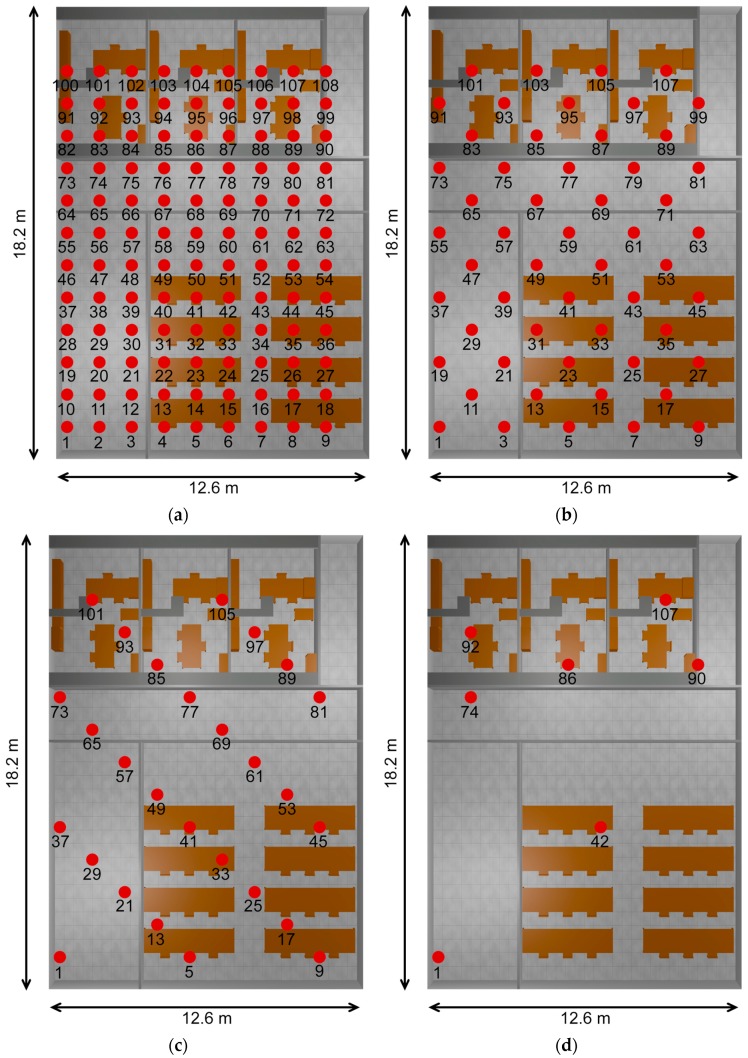
Transceiver Location within the scenario as a function of transceiver node density (**a**) 108 transceivers, (**b**) 54 transceivers, (**c**) 27 transceivers, (**d**) 7 transceivers.

In order to analyze wireless channel performance in complex indoor scenario with variable node density, a variable number of nodes have been embedded in the scenario previously depicted in Figure 1. Node density has varied from 1 transceiver/room to 1 transceiver/2 m^2^ following both uniform and random distributions. The resulting network configurations are schematically depicted in Figure 2. In the case of uniform distributions, symmetrically deployed sensors are activated or deactivated as a function of operating conditions. Modelling of ad-hoc network configurations has been taken into account by employing random distributions of the nodes, by obtaining a random numbering sequence of the active transceivers present in the initial high density uniform scenario, which has 108 transceivers in an overall surface of 216 m^2^. Different layout configurations have been depicted in Figure 2. The proposed density variations allow considering multiple operation scenarios, such as conventional user connectivity (e.g., access with mobile terminals or laptops to WLAN networks, which have initially a low transceiver density, to communication enabled environments within IoT, in which a high node density, owing to embedded transceivers in elements such as furniture can be considered). Transceiver node locations have been considered following three specific configurations: uniform distribution (in which nodes are all equidistant one to another, corresponding to the case of static nodes, representing for example infrastructure nodes), random distribution (in which a sub-set of the maximum density uniform distribution is estimated following a random sequence generator) and a set of wearable nodes, corresponding to extensions given by Wireless Personal Area Networks that can be operating within the scenario.

The lowest node density corresponds to the initial scenario (e.g., previous to IoT enabling technologies) in which one node per each room is placed, corresponding to a non-uniform node distribution. The equivalent node density in terms of surface unit would be: Office Rooms (nodes #86, #92, #107) 1 transceiver/18 m^2^, upper right hallway (node #90) 1 transceiver/9 m^2^, middle hallway (node #74) 1 transceiver/22 m^2^, lower left hallway (node #1) 1 transceiver/36 m^2^ and lab facilities (node #42) 1 transceiver/95 m^2^.

Simulation results have been obtained by application of the 3D Ray Launching code, in which full consideration of the indoor furnishings (shape, volume and dispersive material parameters) is employed. Results have been obtained for the complete scenario volume, for frequencies of 2.4 GHz and 5.8 GHz. Bi-dimensional received power distribution levels have been depicted in order to provide a comprehensive description on the evolution of received power levels, which has direct impact on link balance as well as potential interference levels within the scenario. Results have been obtained for uniform node distributions, with the following parameters:

### Total Surface of Test Scenario: 216 m^2^

-Uniform Distribution:

- 7 antennas (1 transceiver/room): 1, 42, 74, 86, 90, 92, 107.
- 27 antennas (1 transceiver/8 m^2^): 1, 5, 9, 13,…, 105. (numbering scheme *n* + 4)
- 54 antennas (1 transceiver/4 m^2^): All odd-numbered nodes.
- 108 antennas (1 transceiver/2 m^2^): All nodes except wearables

**Figure 3:**
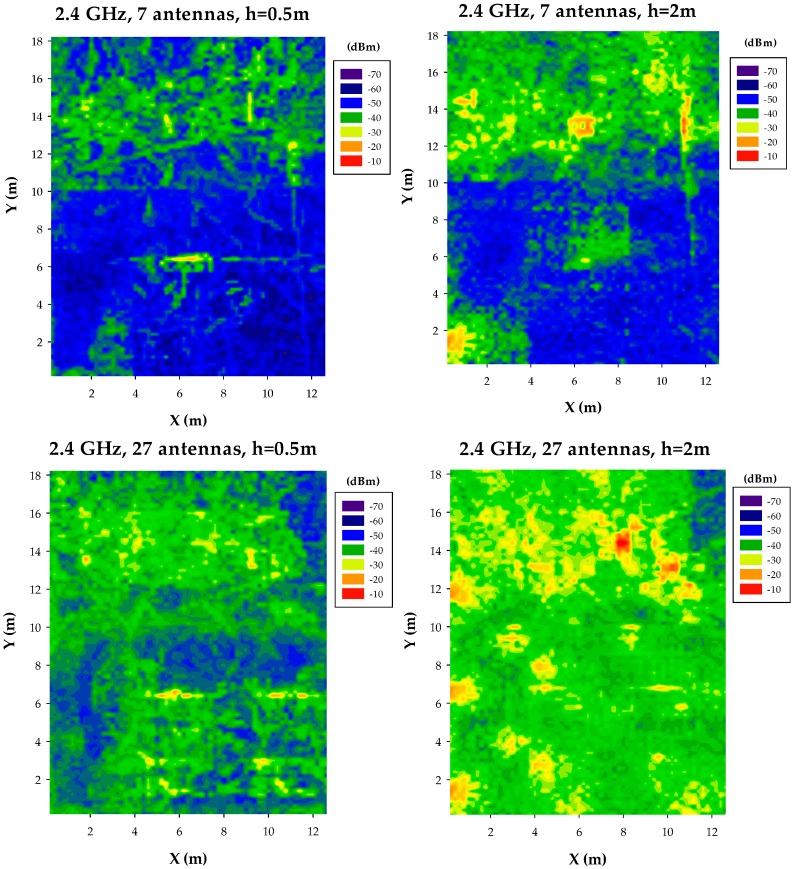
Estimation of received power levels, for an operating frequency of 2.4 GHz, for a scenario with 7 nodes and 27 nodes, at bi-dimensional planes at a height of 0.5 m and 2 m, respectively.

**Figure 4:**
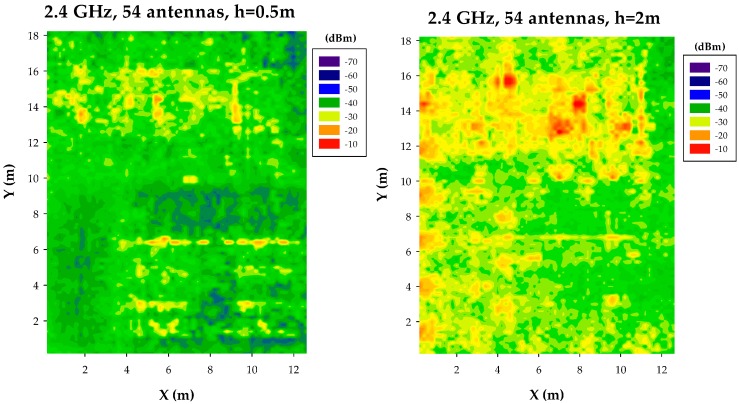

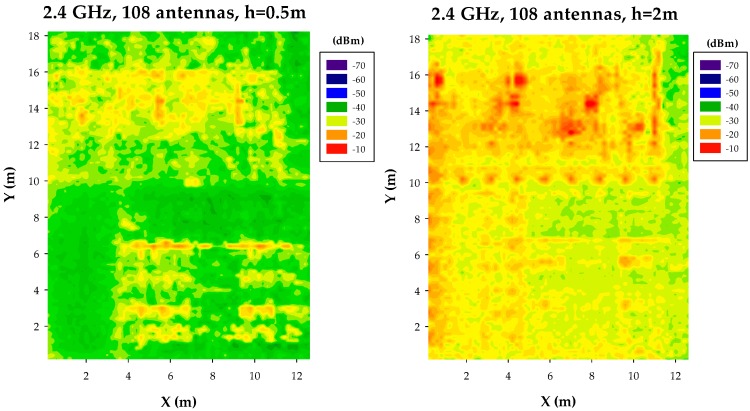
Estimation of received power levels, for an operating frequency of 2.4 GHz, for a scenario with 54 nodes and 108 nodes, at bi-dimensional planes at a height of 0.5 m and 2 m, respectively.

The results for the different node densities are depicted in Figure 3 for the case of seven nodes/scenario and 27 nodes/scenario and in Figure 4 for 54 nodes/scenario and 108 nodes/scenario, in both cases for bi-dimensional received power planes at 0.5 m and 2 m height, respectively. Results highlight that node distribution significantly modifies received power levels, as a function of node density (with values ranging from approximately −50 dBm in the case of seven nodes/scenario to −30 dBm in the case of 108 nodes/scenario) and node location (node height provides additional power variation in the order of 5–10 dB). It is worth noting that individual transceivers lead to localized regions with higher power levels, i.e., hot-spots, within the scenario, which convey into power clusters as the number of nodes increases. This results is relevant in terms of interference analysis, as well as on the operation of mitigation schemes, such as power handling or distributed routing algorithms/aggregation schemes. Node density variation also impacts on deviation of received power levels, with values in order of 20 dB for the low density case to lower than 10 dB in the high density case (excluding the vicinity of hot-spots). Moreover, different zones can be identified within the simulation scenario, in which power distributions differ, such as upper region and lower region. These zones are delimited by the boundaries of the scenario, given in this case by the separation walls between office zones (upper part of the scenario), hallway (middle part) and lab zone (lower part). The existence of such boundaries, inherent in indoor scenarios, gives rise to different effects in terms of wireless channel behavior, such as highlighting the presence of hot-spots (clearly visible at cut-planes which are closer to the transmitter sources, for all of the proposed node densities) and increasing path loss values, given the increase in losses mainly due to absorption and scattering. 

It is also worth noting that power level distributions tend to be more uniform as the number of transceivers increases, which is a direct consequence of the fact that as node density increases, propagation mechanisms will be mainly driven by Line of Sight operation. In this way, transceiver nodes will tend to have similar behavior, with higher average received power levels in the potential communication links that can be established. This is however strongly influenced by the communication scheme employed, since received power level distribution profiles also hold for the potential interference also sources (which also potentially increase, especially in the case of intra-system and inter-system interference), which will increase as node density increases.

In terms node density variation, when transceiver locations are uniformly spaced, power level distributions tend to be more uniform, given that potential receivers can be equidistant to embedded transceivers within the scenario and hence, link balances tend to be similar for all of those potential receivers. As node density increases, path loss tends to decrease, which (if no interference is considered) provides higher values of received power. This fact has also a direct impact in terms of overall interference values, which is a key point in system level analysis, as transmitted power from those nodes that are not in the established communication link contribute as intra-system or inter-system interference, leading to system performance degradation, as will be shown later on in Section 4.

**Figure 5:**
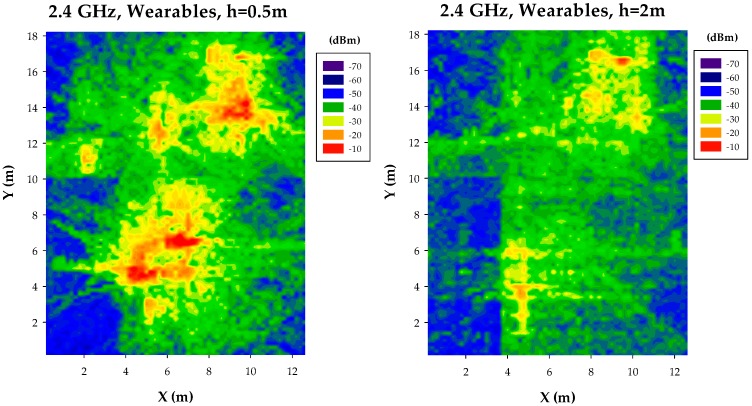
Estimation of received power levels, for an operating frequency of 2.4 GHz, for the case of ad-hoc wearable transceiver configuration.

In the case of Context Aware scenarios, on-body transceivers corresponding to wearables can also be operating, independently or in a collaborative scheme with other transceivers. To take into account these cases, a sub-network of wearable devices has also been implemented and simulated. The results are depicted in Figure 5. In this case, the 2D distributions of received power levels show that hot spot identification is more apparent in the case of a cut-plane height of 0.5 m. This condition is given by the fact that wearables have been located within the upper half of a conventional human body model (e.g., consideration of wrist-worn devices). An additional consequence of employing wearable transceivers is the existence of hot-spots in the vicinity of each wearable node. This fact is relevant in terms of potential interference generated by these nodes, which is time-dependent and will affect infrastructure nodes as well as other users (non-wearable as well as wearable nodes). Therefore, simplified received power estimations, considering average losses owing to transceiver typology (i.e., infrastructure, mobile, wearable) provides limited insight both in terms of received power level compared with required receiver sensitivity or overall interference.

The previous results have been obtained by deploying a uniform set of transceivers within the scenario. In order to provide more realistic cases (mainly considering time dependent links, such as mobile terminals provided by users) non uniform distributions have also been considered. A test case with a total of 10 transceivers has been considered, in order to clearly visualize topological differences in relation with the different configurations. In this case, a higher level of spatial sparseness aids in the process of discriminating the different cases. The following transceivers have been used (obtained by random number generator outputs), forming a subset of elements from the initial complete set given by the uniform network of highest density:

- Random Transceiver Set1: 15, 22, 36, 46, 59, 64, 68, 82, 88, 104.
- Random Transceiver Set2: 5, 8, 29, 32, 43, 46, 65, 92, 98, 105.
- Random Transceiver Set3: 21, 25, 26, 39, 55, 69, 78, 83, 98, 108.

**Figure 6:**
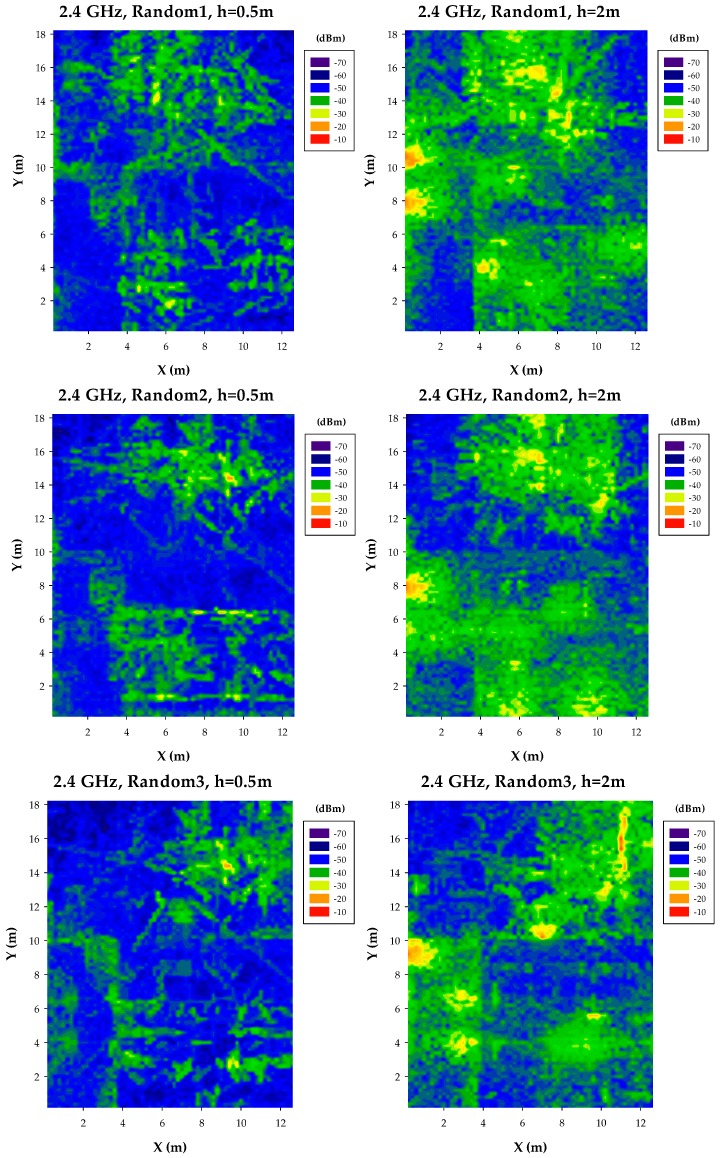
Estimation of received power levels, non-uniform distribution, for a frequency of 2.4 GHz, at bidimensional planes at a height of 0.5 m and 2 m, respectively.

The results for the three random transceiver sets are depicted in Figure 6, for different cut plane heights (0.5 m and 2 m, respectively). As expected, variations in power distribution profiles are larger than in the uniform case. Even though the number of transceiver is the same, power distribution levels are considerably different, since both non-line of sight and line of sight schemes can be emphasized, given the relative distance between nodes for each of the transceiver sets. In the case of random distributions depicted, non-uniformity can give rise to high node concentration, leading to the location of hot-spots within the scenario. Two of these hot-spots can be identified in Figure 5, for a cut-plane height of 0.5 m, in which power distribution profiles are considerably different than in the uniform cases previously described. These hot-spots can strongly degrade system performance, as signal to noise ratios tend to decrease significantly as node density increases, with spatial power deviation in the order of 10–20 dB for distances within conventional transceiver link operation.

**Figure 7:**
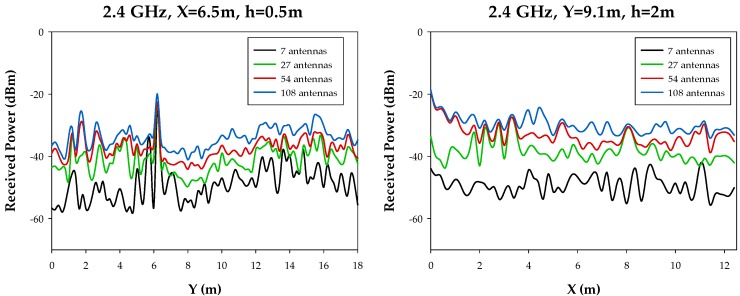
Linear received power level distribution along a longitudinal radial (**left figure**) and a transverse radial (**right figure**), for a frequency of 2.4 GHz.

In order to gain insight on the effect of node density variation, linear received power level distributions have been compared and are shown in Figure 7. As it can be seen, average power levels exhibit a variation in the order of 20 dB between the low density cases (i.e., seven nodes/scenario) and the high density cases (108 nodes/scenario). In the vicinity of transmitter nodes, all power level profiles converge, given that nodes transmit at nominal output power. It is worth noting that the average received power level estimations can also be seen as the equivalent noise level (in terms of intra-system, i.e., users within the same network or inter-system if they are in different networks), which increase as the number of nodes increase. Therefore, in terms of coverage analysis, results improve as the number of nodes increases and in terms of system performance, the equivalent noise floor rise must be considered in order not to degrade potential communication links.

**Figure 8:**
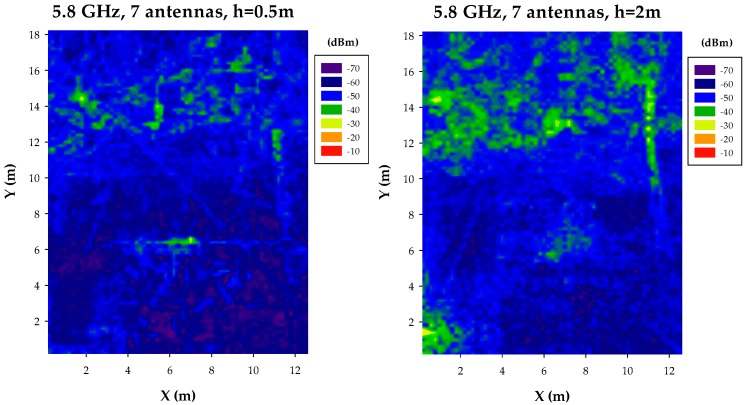

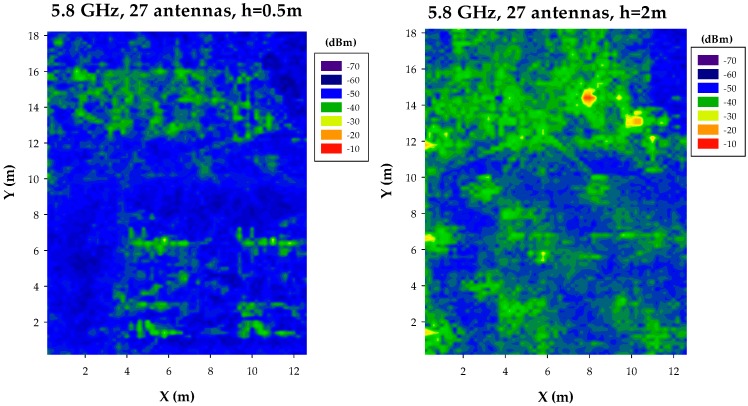
Estimation of received power levels, for a frequency of 5.8 GHz, for a scenario with 7 nodes and 27 nodes, at bi-dimensional planes at a height of 0.5 m and 2 m, respectively.

**Figure 9:**
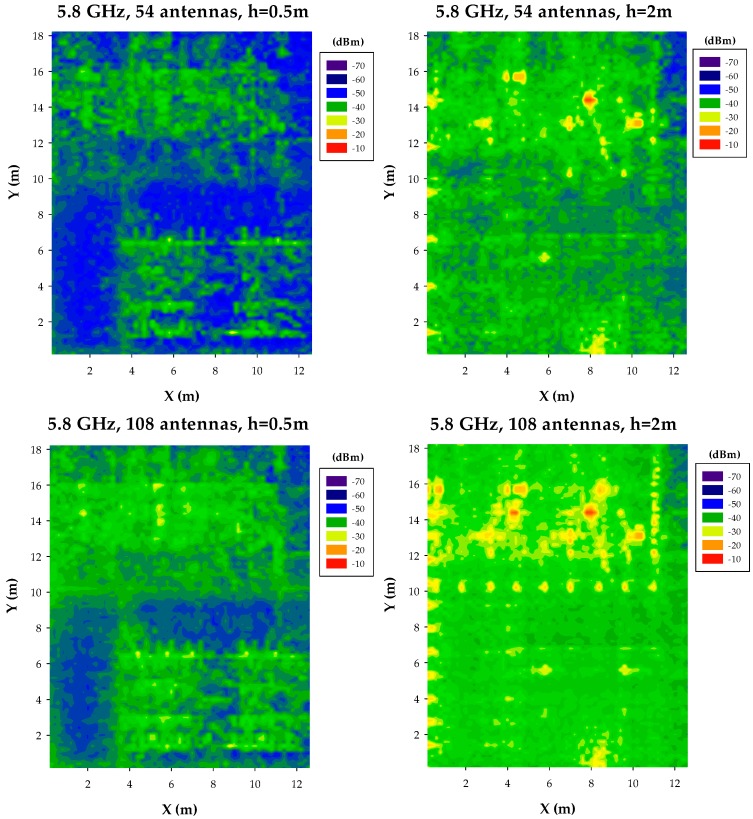
Estimation of received power levels, for a frequency of 5.8 GHz, for a scenario with 54 nodes and 108 nodes, at bi-dimensional planes at a height of 0.5 m and 2 m, respectively.

**Figure 10:**
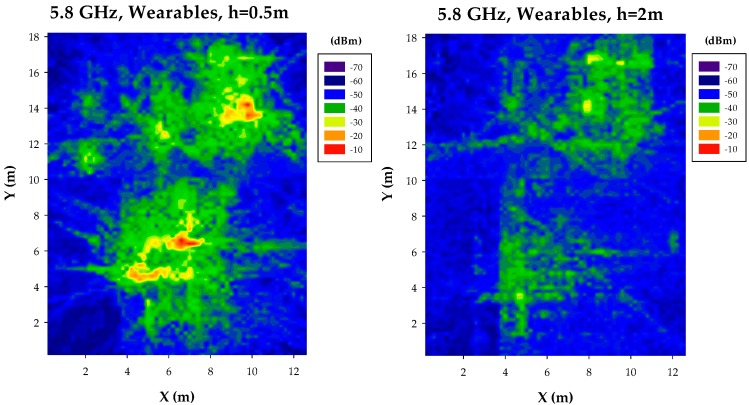
Estimation of received power levels, for a frequency of 5.8 GHz, for the case of ad-hoc wearable transceiver configuration.

Estimations have also been obtained for the case of using a frequency of 5.8 GHz. The change in frequency implies higher attenuation given the use of a smaller wavelength, which can be seen in the results presented in Figure 8, Figure 9 and Figure 10 for the case of uniform network layout, with variable node density values. Power distribution is also different within the bi-dimensional planes, given the fact that wave/obstacle interaction is also modified within the ray launching scheme, given once again by the geometrical changes inherent to smaller operational wavelength. 

**Figure 11:**
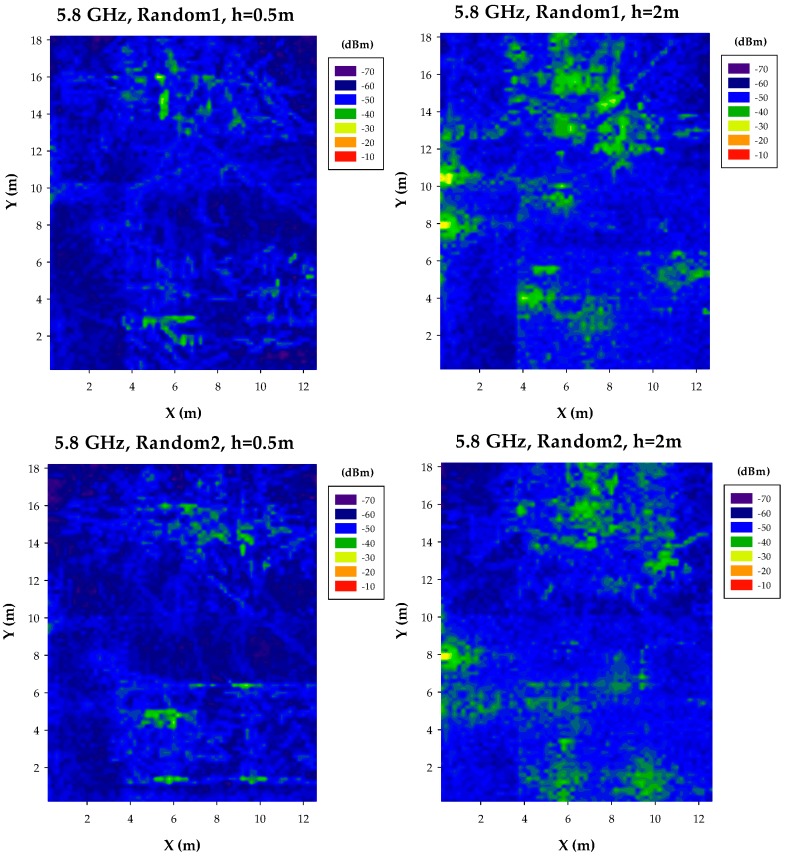

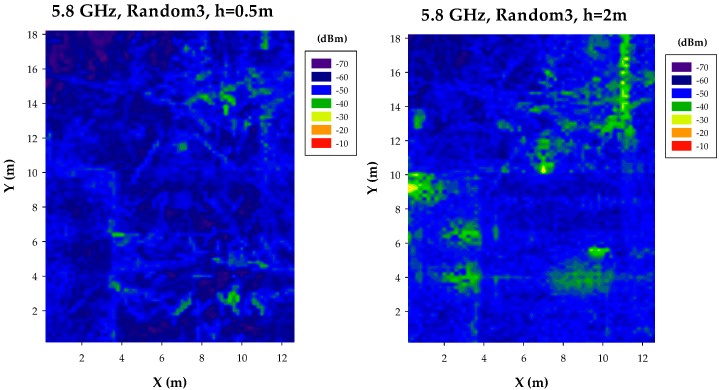
Estimation of received power levels, non-uniform distribution, for a frequency of 5.8 GHz, at bidimensional planes at a height of 0.5 m and 2 m, respectively.

Non-uniform random distributions of transceivers have also been considered for the case of 5.8 GHz, depicted in Figure 11. As with the uniform cases, received power levels are lower, which modifies link balance levels as well potential interference values (intra-system interference contribution, mainly). Provided that due to lower transceiver count in the case of random node sets employed and higher path losses within the 5.8 GHz frequency band, hot-spot identification is also less defined.

**Figure 12:**
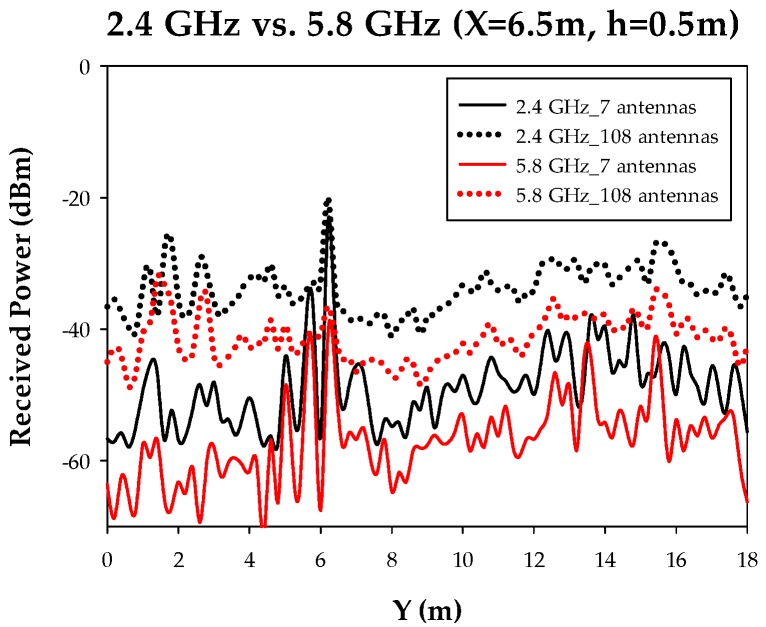
Linear received power level distribution for a frequency of operation of 2.4 GHz vs. 5.8 GHz.

In order to highlight the impact of frequency selection within the 2.4 GHz and 5.8 GHz frequency bands, different received power levels have been depicted in Figure 12. As expected, there is a power offset between both frequency bands, owing to inherently higher losses due to frequency increase in the range of 5 dB. The average power levels can be considered as interference level background when performing system level analysis. Therefore, frequency band selection must take into account not only coverage restrictions but also potential noise floor rise given by interference, which impacts on overall system performance.

**Figure 13:**
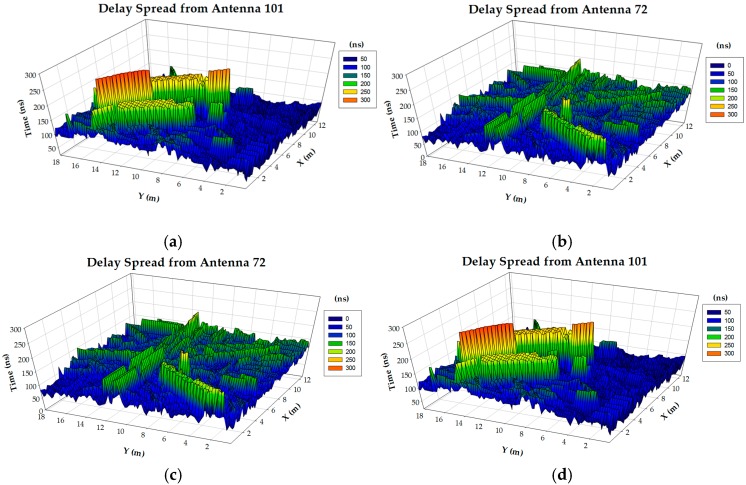
2D Delay Spread distributions for an operating frequency of 5.8 GHz, height plane at h = 2 m for active transmitters: (**a**) TRX #17, (**b**) TRX #60, (**c**) TRX #72, (**d**) TRX #101.

System performance is limited by coverage/capacity relation and hence, precise power level distribution estimations play a key role in QoS analysis. Another relevant parameter to consider is the impact of time-dependent elements, given by multipath propagation and which determine fast fading components. Figure 13 represents bi-dimensional distributions of delay spread (i.e., difference between first and last field component detected at a given transceiver location), considering different potential transceiver nodes. As it can be seen, delay spread distributions are strongly dependent on the transmitting node location and exhibit large variability (Δ_delay-spread_ ≈ 50–150 ns), a direct consequence on the influence of the specific characteristics of the surrounding environment in wireless channel propagation, particularly in the case of multipath components. The analysis of delay spread results as a function of the active transmitter under consideration has been performed for the case of several nodes, which are presented in Table 4. Node locations have been selected in order to provide different cases of observable object density and hence, node-scenario interaction. Average values are similar in all cases (in the order of 115 ns, corresponding to approximately 35 m), with variations in standard deviation values (from 24 ns to 39 ns, corresponding to 7.5 m to 12 m). The average values are determined by the dimensions of the indoor scenario and the conditions for the extinction of reflected rays, whereas the variations in standard deviation are more site-specific, related with the density and location of objects. The use of deterministic analysis can aid in the estimation of both average values and standard deviation, providing a qualitative assessment in terms of node location and density, with site-specific consideration for the particular scenario under analysis. 

In the case of low bit rate transmissions, impact will be low (variations within coherence bandwidth). However, care should be taken in the case of increasing bit rate (vicinity of Gbps capacities in certain future applications linked to ultra-high definition video streaming, virtual or augmented reality, etc.). Delay spread estimations can be then employed in the effective design of dispersion mitigation techniques, such as channel equalization.

## 4. Interference Impact as a Function of Node Density

The number of nodes as well as their topological distribution, in relation with the surrounding environment, determine received power level distributions, which in turn determine effective signal and interference levels. Adequate communication link establishment in terms of quality is provided if the following condition is met:

(1) $$P_{rx}\left(\vec{r},rx-hw\right)\geq SENS_{rx}\left(serv,R_{b},mc\right)$$

where *P_rx_* is the received power for each transceiver, as a function of spatial location *r* and receiver parameters *rx − hw* (e.g., antenna gain. noise factor) and *SENS_rx_* is the receiver sensitivity, determined by the required Signal to Noise Ratio (SNR) threshold (or E_b_/N_o_ in the case of digital systems), transmission bit rate *R_b_* and the modulation and coding scheme *m_c_*. In this context, the determination of useful received power and detected interference levels provides coverage/capacity relations as a function of service requirements and density of intra-system and inter-system users within the scenario. The location of all transmitting as well as receiving elements within the scenario is a fundamental parameter, due to large variability in power distribution in the indoor environment under consideration.

Once wireless channel characterization has been performed, with the aid of a scalable scenario in terms of transceiver density, as described in Section 3, interference analysis can be obtained, deriving in system coverage/capacity estimations. To this extent, based on these results, SNR volumetric estimations have been obtained, with the following rules:

- At 2.4 GHz, typical low cost devices (such as ZigBee) uses O-QPSK modulation, and a typical low-cost detector implementation is expected to meet the 1% Packet Error Rate (PER) requirement at SNR values of 5 dB to 6 dB (following Annex E of the standard IEEE 802.15.4). The results can be extended to other modulation schemes, as a function of the employed wireless systems.
- SNR minimum ZigBee (BW = 3 MHz) at 250 Kbps = −12.26 dB (following Shannon formulation).
- Worst case conditions in terms SNR analysis are provided when the interconnecting device operates with in-band inter-system interference which is in principle independent from interference control mechanisms in native wireless networks of the devices under operation.

Interference analysis results have been obtained by introducing a test transceiver for multiple cases, in which the initial network transceivers for node density variatons have been used as potential interference sources. 

**Figure 14:**
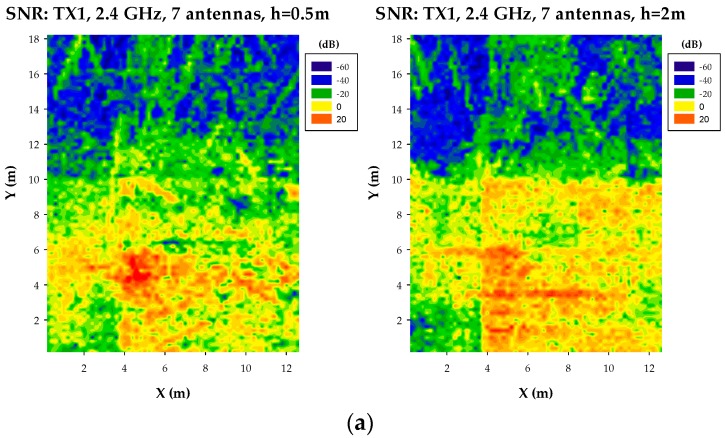

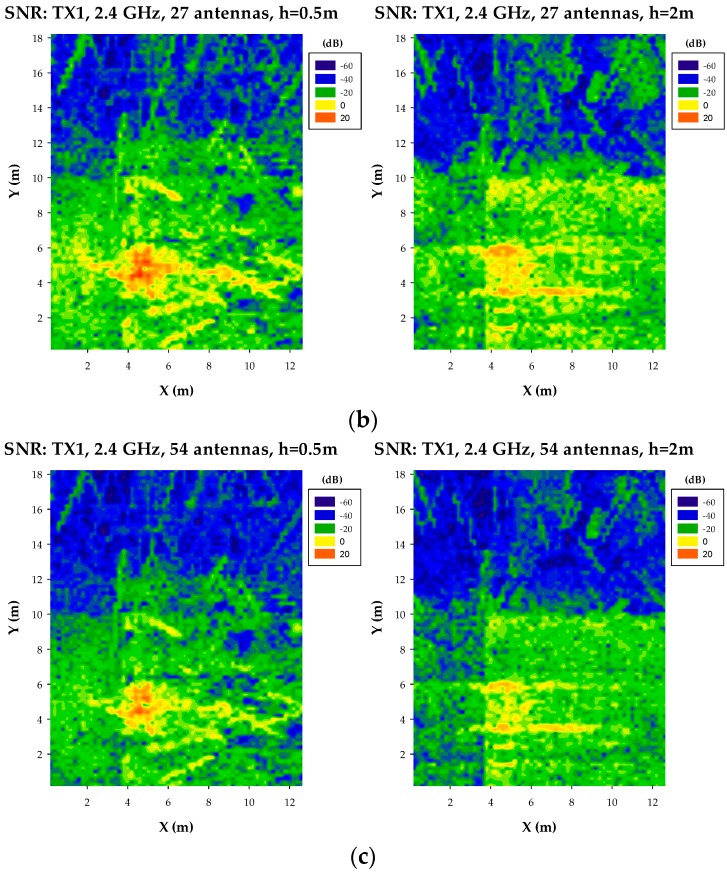

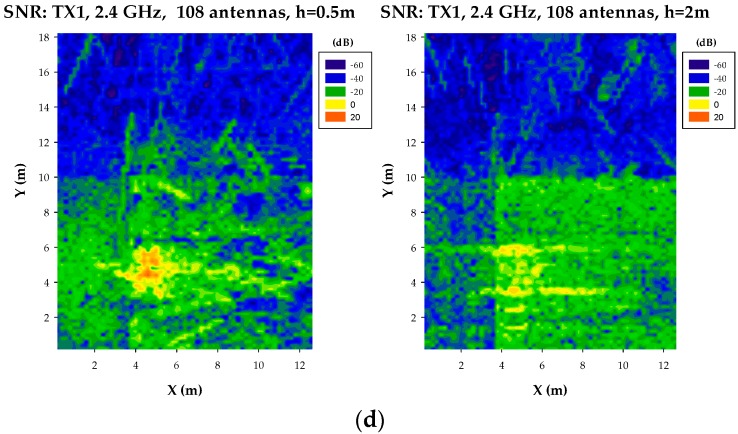
Bi-dimensional distribution of SNR at a frequency of 2.4 GHz, for (**a**) TX1 Wearable A1(109): X = 4.5, Y = 4.5, Z = 1. Interference network: 7 antennas, (**b**) Interference network: 27 antennas, (**c**) Interference network: 54 antennas, (**d**) Interference network: 108 antennas.

**Figure 15:**
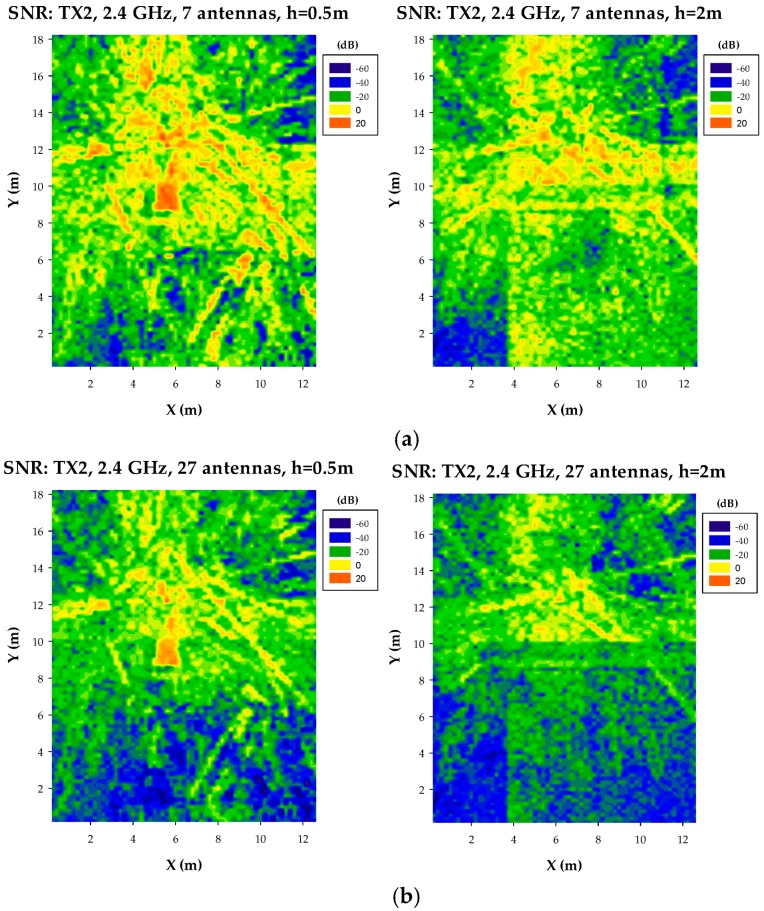

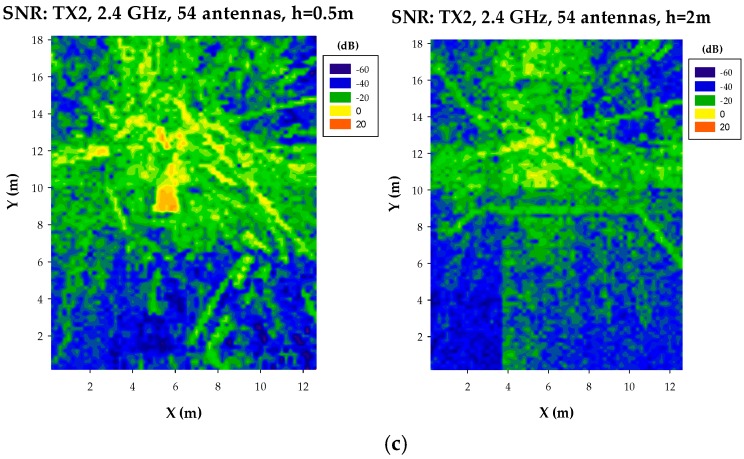

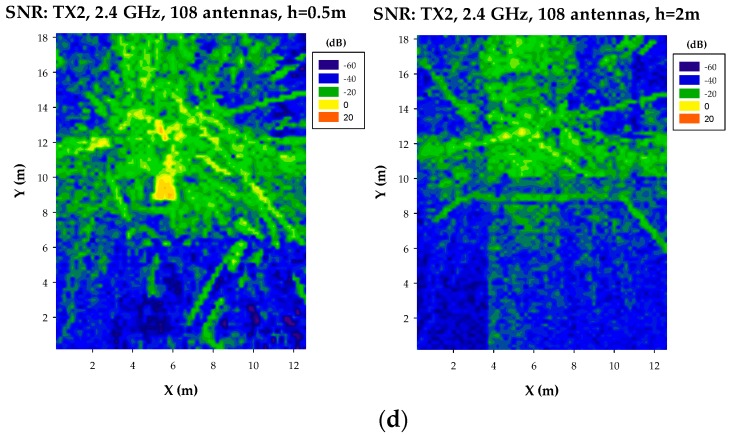
Bi-dimensional distribution of SNR at a frequency of 2.4 GHz, for (**a**) TX2 Wearable D1(118): X = 5.5, Y = 11.7, Z = 1.2. Interference network: 7 antennas, (**b**) Interference network: 27 antennas, (**c**) Interference network: 54 antennas, (**d**) Interference network: 108 antennas.

In this way, results depicted in Figure 14 and Figure 15 show bi-dimensional plots for particular heights of the SNR distribution as a function of potential location of transceivers. These plots represent, therefore, an upper bound in terms of quality degradation in terms of simultaneous operation of other transceivers. The provided SNR values can be mapped afterwards to E_b_/N_o_ ratios, where modulation scheme as well as transmission bit rate can be explicitly considered.

**Figure 16:**
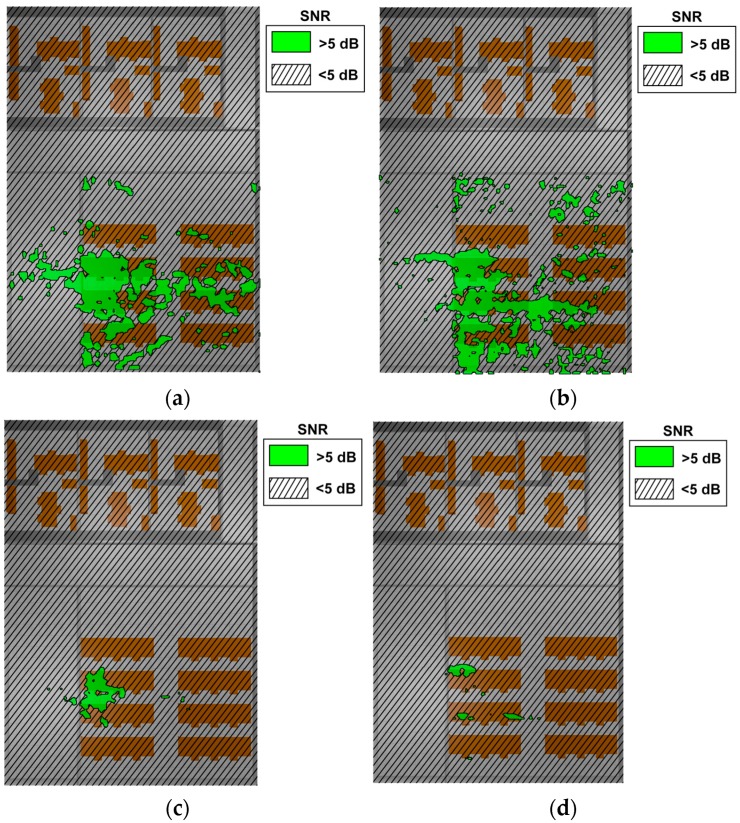

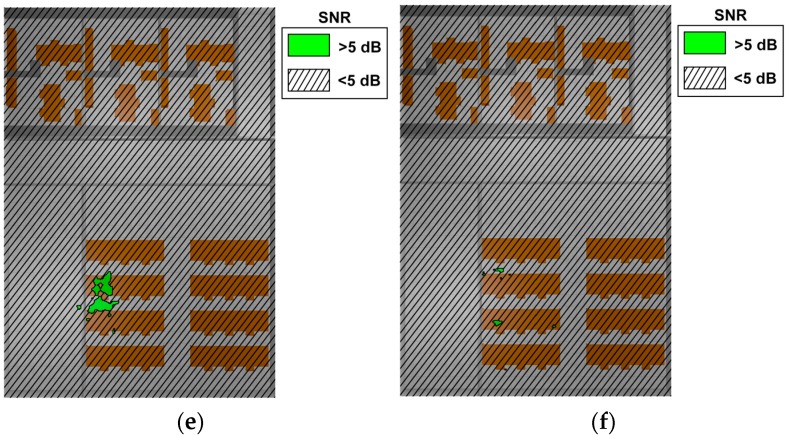
Estimation of bi-dimensional SNR values for the scenario with 7 transceivers (active transmitter TRX1) (**a**) height h = 0.5 m (**b**) height h = 2 m, 27 transceivers (**c**) height h = 0.5 m (**d**) height h = 2 m, 54 transceivers, (**e**) height h = 0.5m, (**f**) height h = 2 m.

Following the rules previously mentioned, one of the possible cases has been depicted in Figure 16, in terms of compliance/non-compliance to a specific SNR threshold, which has been set to a value of SNR >5 dB, consistent with PER <1% for O-QPSK in 802.15.4 standard. Again, results are shown for a given number of interfering nodes introduced in simultaneous operation with other devices, for example wearables. 

**Figure 17:**
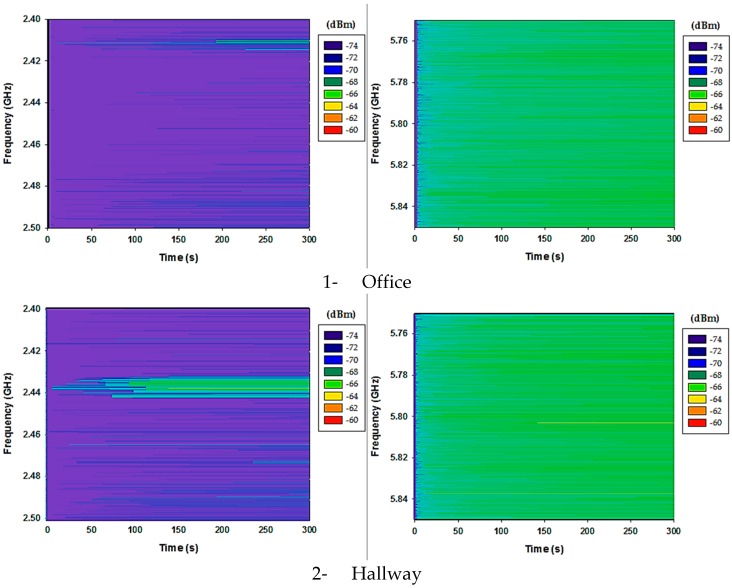

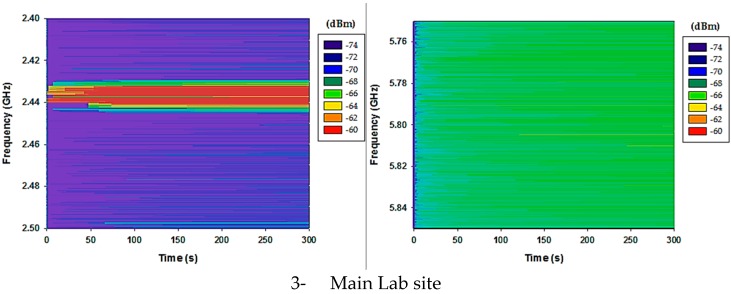
Measured spectrograms for the real test scenarios for 2.4 GHz and 5.8 GHz respectively. Channel occupation is clearly observed within the 2.4 GHz band, whereas noise floor level can be observed in the case of the 5.8 GHz frequency band.

Regions of correct operation can be mapped along the bi-dimensional cut planes (results have been obtained for the complete simulation volume, depicting specific cut-planes for the sake of clarity). These operating regions are once again delimited by the configuration of the interfering network (which is effectively an operating network providing useful signals to another system within the context aware scenario), as well as by the characteristics of the environment. The existence of higher noise floor levels in the case of higher node density is consistent with the reduction in the operating area within the scenario and hence, resulting in a negative impact in overall system operation. In relation with interference analysis, it is worth noting that background noise floor levels have been measured with the aid of a FieldFox spectrum analyzer. The measured spectrograms for the three different zones in which the scenario is divided (i.e., Office, Hallway and Lab zones) are presented in Figure 17. 

The measurement setup employed an ACA-4HSRPP-2458 antenna from ACKme Networks (gain@5 GHz is 3.5 dB larger than gain@2.4 GHz, increasing overall noise floor levels) and a resolution bandwidth of ResBW = 10 KHz. In the case of 2.4 GHz, detected power levels are larger in specific bands, owing to inter-system use by the native WLAN implemented within the scenario, whereas in 5.8 GHz levels are all similar, consistent with lower spectral usage in this case. 

## 5. Conclusions and Future Work

In this work, the performance of wireless system operations in terms of radio channel analysis has been presented, for the specific case of variable node densities in indoor context aware environments. A realistic indoor environment has been recreated and simulated with the aid of deterministic 3D Ray Launching code, implemented ad-hoc and optimized by hybrid simulation techniques in order to simulate large, complex scenarios. A fundamental aspect in order to envisage adequate deployment strategies within heterogeneous wireless networks is to consider large densities of transceivers which can coexist in regions with multiple elements, such as furnishings or the presence of users. In this way, node density variation has been analyzed from 1 node/room to 1 node/2 m^2^, with a total of 108 potential transceivers operating simultaneously. Results have been obtained for the complete volume of the scenario and depicted for several bi-dimensional cut planes and for linear radials of received power levels. The results provide the trend in overall received power levels, ranging in averages from −50 dBm to −30 dBm depending on node density. Moreover, the architecture of the network has been considered, by analyzing uniformly distributed nodes as well as subsets of randomly distributed nodes, a realistic consideration particularly in the case of portable devices. Due to the fact that IoT envisages the massive adoption of body area network devices, several wearable transceivers have also been included within the scenario, exhibiting specific patterns in relation with hot-spot identification in the vicinity of the user, as well as the potential degradation in terms of received power levels due to non-line of sight operation. Wireless channel characterization enables not only to estimate received power levels with precision, it also provides information related with interference levels, which can be given by intra-system or inter-system interference, in the case of networks which can coexist within the scenario. Multiple results have been obtained in order to analyze the impact of transceiver location as well as of node density, which can be considered equivalently as an increase in the effective amount of users located within the scenario. The results show a clear influence of the scenario as well as on the negative impact on increased transceiver density, especially if the operate in a non-centralized way, typical in inter-system interference conditions. Both 2.4 GHz and 5.8 GHz frequency bands have been considered, showing different power distributions due to larger propagation losses as frequency is increased, as well as changes in scenario interaction due to modification in object size vs. wavelength. The results give rise to SNR maps, related with specific quality metrics, such as PER thresholds, indicating regions of service compliance/non-compliance, as a function of required QoS and transmission bit rates. Node density evolution as well as non-uniform concentrations lead to hot-spot formation have direct impact on overall system performance, which will be site-specific in quantitative terms but can provide overall considerations qualitatively. The proposed methodology enables accurate estimations of interference effects and hence determine optimal network configuration layouts for high node density scenarios inherent to IoT and context aware environments. Future work is related with increasing scenario case study as well as increasing system level analysis, with parametric studies in relation with QoS specific parameters, such as outage probability.

## Figures and Tables

**Table 1:** Dielectric permittivity (ε_r_) values for the materials employed in the simulation scenarios.

Material	2.4 GHz	5.8 GHz
Concrete	8.1	5.5
Brick Wall	4.44	3.56
Glass	6.06	5.98
Wood	2.88	2.05
Plasterboard	2.02	2.02

**Table 2:** Conductivity values for the materials employed in the simulation scenarios (S/m).

Material	2.4 GHz	5.8 GHz
Concrete	0.02	5.01 × 10^−2^
Brick Wall	0.11	9.46 × 10^−2^
Glass	0.11	2.99 × 10^−1^
Wood	0.21	8.23 × 10^−2^
Plasterboard	0	1.48 × 10^−2^

**Table 3:** 3D-Ray launching simulation parameters.

Parameter	2.4 GHz	5.8 GHz
Transmitted power	10 dBm	10 dBm
Transmission data rate	250 Kbps	1 Mbps
Antenna type	Monopole	Monopole
Antenna gain	0 dB	0 dB
Launched rays angle resolution	1°	1°
Maximum permitted reflections	6	6
Cuboids resolution	20 cm	20 cm

**Table 4:** Delay Spread values at 2 m height.

Antenna Number (#)	Mean Value (ns)	Standard Dev (ns)
17	110	31
23	117	31
60	123	26
65	120	35
72	111	24
101	105	39
